# Characteristics, practice patterns, and outcomes in patients with acute hypertension: European registry for Studying the Treatment of Acute hyperTension (Euro-STAT)

**DOI:** 10.1186/cc10551

**Published:** 2011-11-16

**Authors:** Alain Vuylsteke, Jean-Louis Vincent, Didier Payen de La Garanderie, Frederick A Anderson, Leigh Emery, Allison Wyman, Sophie Rushton-Smith, Joel M Gore

**Affiliations:** 1 Department of Anaesthesia & Intensive Care, Papworth Hospital NHS Foundation Trust, Papworth Everard, Cambridge, CB23 3RE, UK; 2 Department of Intensive Care, Erasme University Hospital, Route de Lennik 808 1070, University of Brussels, Brussels, Belgium; 3 l'hôpital Lariboisière, 2 Rue Ambroise Paré, 75475 Paris Cedex 10 Assistance publique-Hôpitaux de Paris (AP-HP), Université Paris Diderot-Paris 7, Paris, France; 4 Center for Outcomes Research, University of Massachusetts Medical School, One Innovation Drive, Suite 110 Worcester, MA 01605 USA; 5 Department of Quantitative Health Sciences, University of Massachusetts Medical School, 55 Lake Avenue North, Worcester, MA 01655 USA

## Abstract

**Introduction:**

Although effective strategies are available for the management of chronic hypertension, less is known about treating patients with acute, severe elevations in blood pressure. Using data from the European registry for Studying the Treatment of Acute hyperTension (Euro-STAT), we sought to evaluate 'real-life' management practices and outcomes in patients who received intravenous antihypertensive therapy to treat an episode of acute hypertension.

**Methods:**

Euro-STAT is a European, hospital-based, observational study of consecutive adult patients treated with intravenous antihypertensive therapy while in the emergency department, perioperative unit or ICU. Enrolment took place between 1 July and 15 October 2009 in 11 hospitals in 7 European countries (Austria, Belgium, Germany, Italy, Spain, Sweden and the United Kingdom).

**Results:**

The study population was composed of 791 consecutive patients (median age 69 years, 37% women). Median arterial blood pressure before treatment was 166 mmHg systolic blood pressure (IQR 141 to 190 mmHg) and 80 mmHg diastolic blood pressure (IQR 68 to 95). Nitroglycerine was the most commonly used antihypertensive treatment overall (40% of patients), followed by urapidil (21%), clonidine (16%) and furosemide (8%). Treatment was associated with hypotension in almost 10% of patients. Overall 30-day mortality was 4%, and new or worsening end-organ damage occurred in 19% of patients.

**Conclusions:**

High blood pressure requiring intravenous therapy is currently managed with a variety of agents in Europe, with those most commonly used being nitroglycerine, urapidil and clonidine. Patients with acute hypertension have substantial concomitant morbidity and mortality, and intravenous antihypertensive treatment is associated with hypotension in almost 10% of cases.

## Introduction

Over 1 billion people worldwide are estimated to have hypertension [1,2], which increases their risk of cardiovascular morbidity and mortality [3,4] and organ injury [5,6]. One percent of all hypertensive patients experience at least one episode of acute, severe hypertension, necessitating intravenous (IV) antihypertensive therapy and placing these patients at risk of overt, acute end-organ damage over their lifetime. The actual prevalence may in fact be much higher [7,8]. One-fourth of patients who present to busy emergency departments (EDs) have acute, severe hypertension [7,8]. In the present study, we defined this population by using a pragmatic approach according to whether the treating physicians decided to use IV antihypertensive therapy.

Multiple effective strategies are used for the management of chronic hypertension [1], but less is known about treating patients who present with acute, severe elevations in arterial blood pressure (BP). The optimal target for BP lowering has yet to be defined; the definitions of hypertensive urgencies, emergencies and outcome measures lack uniformity; evidence supporting the use of one drug over another is lacking; and whether certain treatments are better than others is unknown [7]. As a consequence, treatment practices vary [9]. The European registry for Studying the Treatment of Acute hyperTension (Euro-STAT) was conducted to evaluate 'real-life' management practices and outcomes in patients treated with IV antihypertensive therapy for an episode of acute hypertension.

## Methods

### Study design

Euro-STAT is a European, hospital-based, observational study of consecutive patients treated with IV antihypertensive therapy while in the critical care setting. Enrolment took place between 1 July and 15 October 2009 in 11 hospitals in 7 European countries (Austria, Belgium, Germany, Italy, Spain, Sweden and the United Kingdom).

The study sites were selected to include a mix of public and private hospitals across a broad geographic area to ensure a rich and diverse population of patients (Table 1). Participating sites had institutional research board approval for the study and adhered to local regulatory and ethical rules. Patient consent was waived in Austria, Belgium, Germany, Italy, Sweden and the United Kingdom, and oral patient consent was required in Spain. The registry was conducted in accordance with European Union directives on the protection of human subjects in research, local ethical guidelines and the Guidelines for Good Epidemiological Practice http://www.ieaweb.org/index.php?view=article&catid=20:good-epidemiological-practice-gep&id=15:good-epidemiological-practice-gep&format=pdf&option=com_content&Itemid=43. The patients provided their written informed consent to participate if required to do so by local ethical committees. As Euro-STAT is an observational registry, no specific treatments, tests or procedures were mandated or withheld from the patients.

**Table 1:** Sites and investigators participating in Euro-STAT

Country	Site	National coordinator/investigator
Austria	AKH General Hospital	Michael J Hiesmayr
Belgium	Erasme University Hospital	Jean-Louis Vincent
Germany	Universitätsklinik Bonn	Andreas Hoeft
Germany	Charité-Universitätsmedizin	Claudia Spies
Italy	Università degli Studi di Firenze	A Raffaele De Gaudio
Italy	Sant'Andrea Hospital	Salvatore Di Somma
Spain	Hospital de Sabadell	Antonio Artigas
Sweden	Karolinska Universitetssjukhuset, Solna	Jan Ostergren
United Kingdom	New Cross Hospital Wolverhampton	Giampaolo Martinelli
United Kingdom	Papworth Hospital NHS Trust	Alain Vuylsteke
United Kingdom	Southampton General Hospital	David Smith

Euro-STAT, European registry for Studying the Treatment of Acute hyperTension; NHS, National Health Service.

### Study objectives

The objectives of the registry were (1) to improve the understanding of the characteristics of patients with acute BP elevation cared for in EDs, perioperative units and ICUs; (2) to describe contemporary IV management practices, including the timing of treatment initiation, medications used to control BP within selected ranges and the time required to achieve BP control; and (3) to determine in-hospital and postdischarge outcomes as they relate to the management of an episode of acute hypertension. A preliminary goal was to establish the rates of key variables, which could then be used to develop formal sample size estimates for a planned larger study in the same setting.

### Patient selection and enrolment criteria

Consecutive adult male and female patients at least 18 years of age with a qualifying episode of acute hypertension on or after 1 July 2009 were identified by trained data abstractors through prospective identification of patients admitted to EDs, perioperative units and ICUs. To qualify for enrolment, a patient had to meet the following two criteria: (1) The clinician had to have attempted BP control with either an IV infusion or at least two IV boluses of an antihypertensive drug, and (2) the initial treatment had to have been started within 24 hours of the patient's arrival in the ED, at any time during the perioperative period (defined as the time from the initiation of anaesthesia to discharge from the postanaesthesia recovery area) or within 14 days of ICU admission.

Patients were excluded from participating in the study if they had received IV antihypertensive treatment initiated in a transferring hospital, if they were admitted for burn treatment, if they had drug-induced (iatrogenic) hypertension, if the qualifying event had occurred while being treated with comfort measures only, and if medical records were not available. ED patients were excluded if they were transferred directly from the ED to another healthcare facility after initiation of IV antihypertensive treatment. Perioperative patients were excluded if they had planned perioperative controlled hypotension and if the qualifying event occurred before the induction of anaesthesia.

### Data acquisition

All eligible patients were assigned a unique study identification number, and abstractors completed a standardised case report form that had been piloted and subsequently validated. Data were abstracted from hospital medical records.

### Outcome measures

The main clinical outcome measures were all-cause in-hospital mortality, end-organ injury (including ischaemic stroke, encephalopathy (defined as acute mental status changes without other identifiable causes), acute coronary syndrome, congestive heart failure, renal insufficiency ('chronic kidney failure' was defined as previously diagnosed renal insufficiency or documentation of creatinine clearance less than 60 ml/minute before admission) and aortic dissection), and survival at 30 days following hospital discharge.

### Data quality and management

Training material and guidelines for data abstractors were developed by the data-coordinating centre at the Center for Outcomes Research (COR) at the University of Massachusetts Medical School (Worcester, MA, USA). A one-day course was held at each enrolling hospital to maintain common definitions, improve data abstraction and address training needs.

Data were collected, handled and analysed independently of the sponsor by the data-coordinating centre. Study coordinators at COR reviewed the data collection forms to identify variances from norms and missing data and followed up with the principal investigators by mail or by telephone to resolve any queries.

### Statistical analyses

Descriptive statistics were calculated for all patients. Data are summarised as means (± SD) or as medians (25% and 75% percentiles) for continuous data and as counts and percentages for categorical data. All results shown are univariate and unadjusted. No variable had a level of missing data greater than 10%. Any missing data were simply excluded from the analyses. All eligible patients underwent chart review, and there was no loss to follow-up. Patients' charts were reviewed until the patient's death or discharge from the hospital up to 30 days after initiation of IV antihypertensive therapy (whichever came first). Statistical analyses were performed using the SAS version 9.2 software package (SAS Institute, Cary, NC, USA).

## Results

### Overview of study population

The study population comprised 791 consecutive patients with a median age of 69 years, and 37% were women (Table 2). In the overall cohort, 62% had a history of hypertension, 24% had diabetes mellitus, 14% had undergone previous cardiac surgery (including coronary artery bypass graft or valve surgery) and 12% had had a prior myocardial infarction. Patients who were treated during the perioperative period were the youngest and had the highest numerical rates of peripheral vascular disease, tobacco smoking and alcohol misuse compared with the other patients. Patients admitted to the ED were older than the postoperative and ICU populations. The prevalence of history of hypertension (including prior hospitalisation for hypertension), diabetes, neurological disease or dysfunction, chronic kidney disease and end-stage kidney disease was numerically highest among the ED population. The ICU population had the highest prevalence of cardiac surgery and previous myocardial infarction.

**Table 2:** Baseline characteristics of the study population

Variables	All patients (*N *= 791)	Enrolment setting		
		
		ED (*n *= 180)	ICU (*n *= 382)	Perioperative (*n *= 229)
Median age, years (IQR)	69 (58 to 77)	70 (60 to 79)	69 (59 to 76)	66 (53 to 76)
Females, *n *(%)	295 (37)	94 (52)	107 (28)	94 (41)
Medical history, *n *(%)				
Hypertension	491 (62)	126 (70)	230 (60)	135 (59)
Hospitalisation for hypertension	7 (0.9)	6 (3.3)	0 (0.0)	1 (0.4)
Cardiac surgery	108 (14)	6 (3.3)	71 (19)	31 (14)
Myocardial infarction	96 (12)	19 (11)	56 (15)	21 (9.2)
Diabetes mellitus	189 (24)	49 (27)	88 (23)	52 (23)
Chronic kidney disease	80 (10)	25 (14)	43 (11)	12 (5.2)
End-stage renal disease	18 (2.3)	5 (2.8)	10 (2.6)	3 (1.3)
Neurological dysfunction or disease	91 (12)	29 (16)	45 (12)	17 (7.4)
Peripheral vascular disease	91 (12)	13 (7.2)	47 (12)	31 (14)
Tobacco smoker	176 (22)	30 (17)	83 (22)	63 (28)
Alcohol misuse (≥2 drinks/day)	45 (5.7)	2 (1.1)	23 (6.0)	20 (8.7)
Drug abuse (amphetamines, cocaine, other)	2 (0.3)	1 (0.6)	1 (0.3)	0 (0.0)
Predisposing factors, *n *(%)				
Medication nonadherence (ED)	-	11 (6.1)	-	-
Medications withheld > 12 hours (ICU and perioperative)	-	-	39 (10)	21 (9.2)

ED, emergency department; -, no data.

The overall median (IQR) initial and peak creatinine values were 89 μmol/L (72 to 113 μmol/L) and 103 μmol/L (82 to 146 μmol/L), and these values were highest among patients admitted to the ED and lowest among perioperative patients. The median (IQR) arterial pressure at the initiation of treatment was 166 mmHg for systolic blood pressure (SBP) (141 to 190 mmHg) and 80 mmHg for diastolic blood pressure (DBP) (68 to 95 mmHg) (Table 3). The initial BP values were highest among the ED patients.

**Table 3:** Blood pressure, laboratory values, end-organ damage and outcomes (in-hospital)

Variables	All patients (*n *= 791)	Enrolment setting		
		
		ED (*n *= 180)	ICU (*n *= 382)	Perioperative (*n *= 229)
Median blood pressure, mmHg (IQR)				
Qualifying SBP	166 (141 to 190)	200 (174 to 220)	160 (138 to 180)	160 (140 to 180)
Qualifying DBP	80 (68 to 95)	100 (90 to 110)	73 (64 to 85)	80 (68 to 90)
Median time from qualifying BP to initiation of IV antihypertensive therapy, minutes (IQR)	5.0 (2.0 to 17)	20 (5.0 to 30)	5.0 (1.0 to 15)	5.0 (1.0 to 5.0)
Median time from IV initiation to 10% decrease in SBP, minutes (IQR)	28 (11 to 60)	31 (18 to 87)	30 (12 to 70)	18 (8.0 to 43)
Median laboratory values (IQR)				
Peak troponin	0.03 (0.01 to 0.23)	0.03 (0.01 to 0.12)	0.04 (0.01 to 0.33)	0.03 (0.01 to 0.23)
Peak creatine kinase-MB	33 (9.4 to 88)	5.1 (3.8 to 78)	50 (31 to 152)	38 (24 to 79)
Peak NT-proBNP	910 (295 to 2, 240)	1, 672 (429 to 2, 637)	1, 451 (428 to 2, 709)	359 (242 to 970)
Peak BNP	99 (82 to 132)	116 (91 to 146)	20 (20 to 20)	n/a
Initial creatinine, μmol/L	89 (72 to 113)	94 (78 to 125)	91 (74 to 116)	83 (69 to 102)
Peak creatinine, μmol/L	103 (82 to 146)	119 (99 to 222)	107 (84 to 151)	92 (73 to 122)
End-organ injury^a^, *n *(%)				
Acute coronary syndrome (new)	25 (3.2)	22 (12)	2 (0.5)	1 (0.4)
Previous worsened	10 (1.3)	8 (4.4)	2 (0.5)	0 (0.0)
Acute LV dysfunction/pulmonary oedema (new)	28 (3.5)	19 (11)	7 (1.8)	2 (0.9)
Previous worsened	20 (2.5)	16 (8.9)	4 (1.1)	0 (0.0)
Aortic dissection (new)	5 (0.6)	2 (1.1)	3 (0.8)	0 (0.0)
Previous worsened	2 (0.3)	1 (0.6)	1 (0.3)	0 (0.0)
Encephalopathy (new)	22 (2.8)	8 (4.4)	11 (2.9)	3 (1.3)
Previous worsened	2 (0.3)	1 (0.6)	0 (0.0)	1 (0.4)
Acute kidney failure (new)	36 (4.6)	8 (4.4)	23 (6.0)	5 (2.2)
Previous worsened	19 (2.4)	5 (2.8)	12 (3.1)	2 (0.9)
Stroke (new)	13 (1.6)	8 (4.4)	5 (1.3)	0 (0.0)
Previous worsened	7 (0.9)	3 (1.7)	2 (0.6)	2 (0.9)
Outcomes *n *(%)				
ICU admission	114 (14)	17 (9.4)	19 (5.0)^b^	78 (34)
Hypertension relapse	104 (13)	14 (7.8)	55 (14)	35 (15)

BP = blood pressure; DBP = diastolic blood pressure; ED = emergency department; IV = intravenous; LV = left ventricular; n/a = not applicable; NT-proBNP = N-terminal prohormone of brain natriuretic peptide; SBP = systolic blood pressure. ^a^Categories are not mutually exclusive. ^b^Readmission to ICU.

### Intravenous antihypertensive drugs

**Figure 1:**
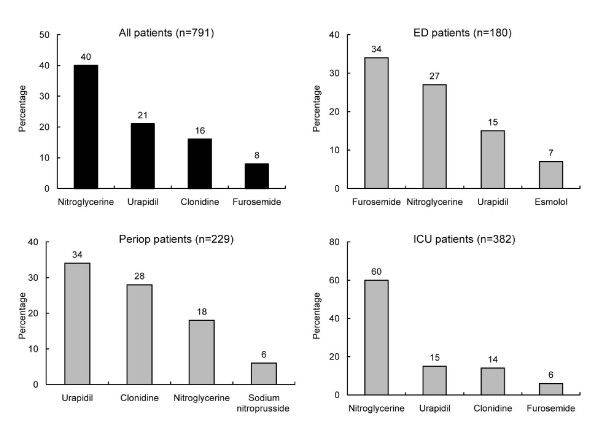
**Most frequent first intravenous antihypertensive drugs used across patient groups**.

The median number (IQR) of IV antihypertensive drugs used during the first three hours was 1 (1 to 2) overall and was similar across the clinical settings: 1 (1 to 2) in ED and perioperative patients and 1 (1 and 1) in ICU patients. The four most frequently used IV antihypertensive drugs in each clinical setting are shown in Figure 1. Nitroglycerine was the most commonly used drug overall (40% of patients), driven largely by its high rate of use among ICU patients (60%). Urapidil and clonidine were the next most frequently used drugs overall, which was due to the high rates of use among perioperative patients. Furosemide was the fourth most often used drug and the most commonly used treatment in the ED population. Among the ED patients whose first IV treatment was nitroglycerine, hypertension was the most common admitting diagnosis (*n *= 16, 33%), followed by acute coronary syndrome (*n *= 8, 16%), chest pain, and heart failure or pulmonary oedema (both *n *= 6, 12%).

In each group, more than 90% of patients achieved a 10% decrease in SBP within the first 72 hours following initiation of IV antihypertensive therapy. The median time to 10% decrease was 28 minutes after starting the antihypertensive drug. The following is the breakdown of patients who did not achieve a 10% decrease within 72 hours: 6 of 180 ED patients (3.3%), 22 of 382 ICU patients (5.8%) and 8 of 229 perioperative patients (3.5%).

### Emergency department patients

**Figure 2:**
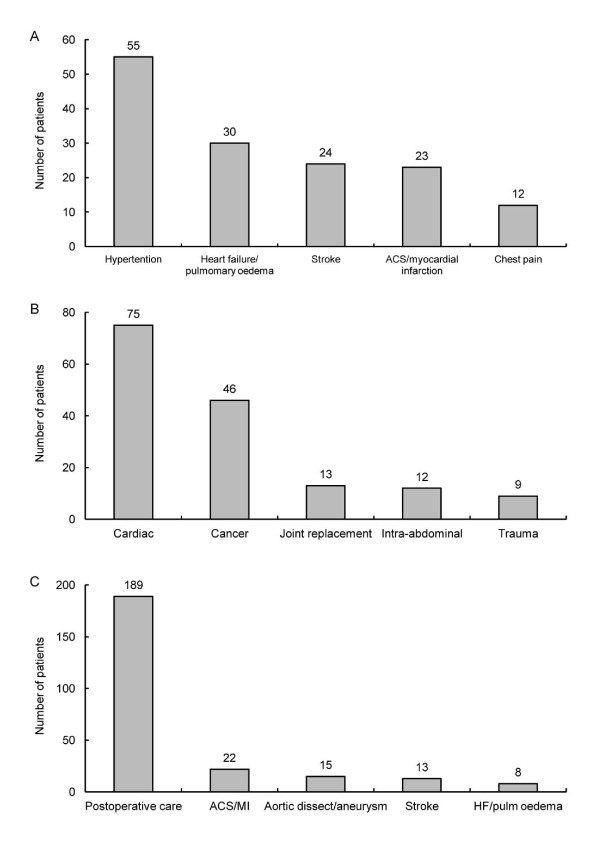
**Presumptive primary admitting diagnoses**. **(A) **Emergency department patients. **(B) **Perioperative patients. **(C) **ICU patients. ACS = acute coronary syndrome; HF = heart failure; MI = myocardial infarction.

Among the 180 patients who presented to the ED, the most common symptoms were shortness of breath (*n *= 60, 33%) and chest pain (*n *= 50, 28%) and, less frequently, headache (*n *= 20, 11%), focal neurological deficit (*n *= 18, 10%) and altered mental status (*n *= 13, 7.2%). The most common presumptive primary diagnoses were hypertension, heart failure or pulmonary oedema, stroke, acute coronary syndrome or myocardial infarction, and chest pain (Figure 2A).

The median (IQR) number of hours in the ED was 8.0 (4.0 to 20), the minimum ED stay was one-half hour and the maximum stay was 144 hours. Most ED patients (*n *= 155, 95%) had their IV antihypertensive treatment started in the ED; 9 patients (5.5%) had it started in the ambulance; 4.4% experienced an episode of hypotension requiring discontinuation of the antihypertensive drug, and/or the administration of vasopressors or fluids, or reverse Trendelenburg positioning within 72 hours of starting treatment; and 3.3% relapsed (requiring resumption of IV antihypertensive treatment) within 72 hours of discontinuation of the initial IV treatment.

The majority of the ED patients were admitted to a non-ICU inpatient unit (*n *= 104, 58%). Fifty-three patients (29%) were discharged to home, 16 (8.9%) were admitted to the ICU, 5 (2.8%) left against medical advice and 2 (1.1%) died in the ED.

### Perioperative patients

The preoperative diagnoses of the 229 perioperative patients are shown in Figure 2B. Most operating or recovery room patients had undergone cardiac (40%), gastrointestinal (17%), orthopaedic (11%) or vascular (9.2%) surgery. The median duration of surgery was 190 minutes. Nearly one-half of the patients (*n *= 112, 49%) had undergone cardiac surgery and were evaluated on the basis of their EuroSCORE (European System for Cardiac Operative Risk Evaluation; available at http://www.euroscore.org/). The median score (IQR) was 2.5 (1.2 to 6.4; minimum 0, maximum 68.6), which is equivalent to a median predicted mortality rate of 2.5% (1.2% to 6.4%). For most patients (*n *= 143, 67%) IV antihypertensive treatment started in the operating room or theatre, and in 33% (*n *= 71) it was begun after surgery. Postoperatively, 57% (*n *= 130) of the patients were transferred to a postanaesthesia recovery area, 31% (*n *= 72) were transferred directly to the ICU and 12% (*n *= 27) were transferred directly to an inpatient ward or unit. Ten percent of patients experienced an episode of hypotension within 72 hours of starting treatment, and 4.8% of patients relapsed within 72 hours of the discontinuation of the initial IV treatment. Thirty-nine percent (*n *= 89) of patients received two or more different IV antihypertensive drugs.

### ICU patients

Most ICU patients (304 of 382, 80%) were direct postoperative admissions (that is, patients whose initial acute, severe hypertension developed after ICU admission). Their primary diagnoses are shown in Figure 2C. The majority of patients in the ICU population were cardiac or cardiac surgery patients (62%), 25% were mixed surgical and medical patients, 7% were surgical patients, 4% were medical patients and 3% were classified as 'other'. Patients spent a median (IQR) of 3 days (2 to 6) in the ICU. Among ICU patients, the minimum stay was 1 day and the maximum stay was 54 days. Thirteen patients (3.4%) died in the ICU. The median time (IQR) in the ICU before IV treatment began was 3.4 hours (0.6 to 18). Overall, 8.4% of ICU patients experienced an episode of hypotension within 72 hours of the start of IV antihypertensive treatment, 6.0% relapsed within 72 hours of discontinuation of the initial IV treatment, and 17% required treatment with more than one agent. Upon discharge, 90% (*n *= 343) of the patients were transferred to an inpatient unit, 3.7% (*n *= 14) were transferred to another healthcare facility, 2.9% (*n *= 11) were discharged to home and 0.3% (*n *= 1) left against medical advice.

### Thirty-day outcomes

The overall rate of 30-day death rate was 4%, and the overall rate of new or worsening end-organ damage was 19%. The rates of both outcomes were highest among patients in the ED (6% and 43%, respectively), intermediate in the ICU patients (5% and 15%, respectively) and lowest among patients in the perioperative setting (2% and 7%, respectively).

## Discussion

These contemporary data derived from the Euro-STAT study demonstrate heterogeneous patterns of care for patients presenting with acute elevation of BP across a range of acute care settings, with high rates of morbidity and mortality. Of the 791 patients included in Euro-STAT, 48% were treated for acute hypertension in the ICU. Many of them had undergone surgery and developed severe hypertension postoperatively, 29% developed it perioperatively and 23% developed it in the ED. Median BP was higher among patients treated in the ED (200/100 mmHg) than in those treated in the ICU (160/73 mmHg) or perioperatively (160/80 mmHg).

The Studying the Treatment of Acute Hypertension (STAT) registry in the United States [9,10] involved patients with a qualifying episode of acute, severe hypertension (that is, (1) SBP greater than 180 and/or DBP greater than 110 mmHg or (2) SBP above 140 mmHg and/or DBP greater than 90 mmHg when presenting with subarachnoid haemorrhage) who received IV antihypertensive therapy in a critical care setting. Compared with the STAT study, the patients' median age in the Euro-STAT study was higher (69 vs 58 years) and fewer patients had a history of hypertension (62% vs 89%). Overall, the median BP was lower in the Euro-STAT study than in the STAT study (166/80 vs 200/110 mmHg). This finding could indicate that patients in the Euro-STAT study had less severe hypertension, or it could be related to the different qualifying criteria and hence the distribution of patients in the STAT studies. In Euro-STAT, perioperative patients were also included, and there were many more ICU patients (mainly in a postoperative context) than there were in the STAT study.

The decision to amalgamate ED, perioperative and ICU patients was made because the purpose of this descriptive study was to evaluate the efficacy of IV therapy for the control of elevated BP level in different locations within a hospital, thereby assessing similarities and differences in treatments and outcomes in different contexts.

The first antihypertensive drug used varied by treatment location. In the ICU, nitroglycerine was by far the most widely used (60%); in the ED, furosemide was used in 34% of patients and nitroglycerine was used in 27%; and perioperatively, urapidil was used in 34% of patients and clonidine was used in 28%. The high use of furosemide in the ED (34%) indicates that pulmonary oedema associated with high BP was the likely primary diagnosis, which is backed up by the higher rates of new or worsened acute coronary syndrome (17% vs 1.1% and 0.5%) and acute left ventricular dysfunction (19.4% vs 2.9% and 0.9%) in ED vs ICU and perioperative patients, respectively. In Euro-STAT overall, the most frequently used antihypertensive drugs were nitroglycerine (40%), urapidil (21%), clonidine (16%) and furosemide (8.3%). This pattern is very different from the results of the STAT study, where the most common initial antihypertensive drugs were labetalol (32%), metoprolol (17%), nitroglycerine (15%) and hydralazine (15%) [9]. The variations in drug use between the Euro-STAT and STAT studies may reflect differences in the drugs available in these regions. The percentage of patients who required a second IV antihypertensive was 34% in both the Euro-STAT and STAT studies.

This variability in treatment between continents, studies and hospital departments is not surprising, given the paucity of evidence regarding the superiority of one drug over another and the absence of guidelines for the treatment of acute hypertension. There are, however, various papers that have provided recommendations that are not evidence-based. Acute hypertension can be split into hypertensive emergencies (severe BP elevation with evidence of impending or progressive end-organ damage) and hypertensive urgencies (severe BP elevation without progressive target organ dysfunction) [1]. In general, hypertensive urgencies can be treated with oral antihypertensive drugs [11], and researchers who published a recent meta-analysis found angiotensin-converting enzyme inhibitors to be superior to calcium channel blockers [12]. Hypertensive emergencies generally require IV treatment to achieve a rapid decrease in BP, and patients admitted to these care settings may be sicker than patients treated with oral agents. Recommendations (based on expert consensus due to a lack of clinical trials) for which antihypertensive to use based on different disease states are shown in Table 4[13].

**Table 4:** Recommended treatments for hypertensive emergencies^a^

Emergency type	Esmolol	Fenoldopam	Labetalol	Nicardipine	Nitroglycerine	Sodium nitroprusside
Pulmonary oedema + systolic dysfunction		Yes		Yes	Yes^b^	Yes
Pulmonary oedema + diastolic dysfunction	Yes		Yes		Yes^b^	Yes
Myocardial infarction	Yes^c^		Yes^c^		Yes^b^	
Aortic dissection	Yes		Yes			Yes
Postoperative hypertension	Yes		Yes	Yes		
Hypertensive encephalopathy		Yes	Yes	Yes		
Kidney failure		Yes		Yes		
Stroke		Yes	Yes	Yes		

^a^Adapted from Smithburger *et al*. [13]. ^b^As adjunctive therapy. ^c^In combination with intravenous nitroglycerine.

While nitroglycerine should be used as an adjunctive therapy, the high rates of use in the Euro-STAT population likely reflect familiarity with its use, together with its ease of administration, titration and rapid reversibility. In the ED, the ICU and perioperatively, 4.4%, 8.4% and 10% of patients, respectively, had hypotension within 72 hours of the start of treatment, resulting in (1) discontinuation of the antihypertensive drug or (2) treatment with vasopressors, fluids or reverse Trendelenburg positioning. The overall incidence of hypotension in the Euro-STAT study was higher than that in the STAT study (8.1% vs 4.0%) [9].

As expected, mortality rates at discharge from the ICU, ED or surgical department in the Euro-STAT study were highest among ICU patients (3.4% vs 1.1% of ED patients and 0% of perioperative patients). Total in-hospital mortality in the Euro-STAT study was highest among ED patients (5.7% vs 5.1% of ICU patients and 2.3% of perioperative patients), which is lower than the comparable rate in the STAT study (6.9%) [9]. New or worsened end-organ damage was observed in 43% of ED patients, 15% of ICU patients and 6.6% of perioperative patients.

### Limitations

The observational Euro-STAT registry is a relatively small data set, and the sites may not be representative of all hospitals in Europe. Treatment choices may therefore be biased by institution choices. Also, populations of acute hypertension are heterogeneous and are likely to differ by country. Amalgamation of all countries into one data set therefore gives an overview, but no country-specific data. As our entry criteria required the physician to initiate IV antihypertensive therapy, there was no comparison with other patients in relation to morbidity. Also, we do not have complete information on post-hospital discharge follow-up. Furthermore, we do not know what happened to patients for whom a reduction in BP was not achieved despite treatment.

## Conclusions

Acute hypertension is currently managed with a wide range of IV agents in the various clinical settings in which it arises. These patients have substantial concomitant morbidity and mortality, and IV antihypertensive treatment is associated with hypotension in almost 10% of patients. Further data are required to identify optimal management strategies for these patients, thereby ensuring the best possible outcomes.

## Key messages

• High blood pressure requiring intravenously administered drug therapy is currently managed with a variety of agents in Europe, with the most commonly used being nitroglycerine, urapidil and clonidine.

• Intravenous antihypertensive treatment is associated with hypotension in almost 10% of patients.

• Patients with acute, severe hypertension have substantial concomitant morbidity and mortality.

## Abbreviations

BP: blood pressure; COR: Center for Outcomes Research; ED: emergency department; Euro-STAT: European registry for Studying the Treatment of Acute hyperTension.

## Competing interests

The Euro-STAT study was supported by a grant from The Medicines Company to the COR at the University of Massachusetts Medical School. All authors have connections to The Medicines Company (Parsippany, NJ, USA). All authors were either employees of, or consultants to, the Center for Outcomes Research, University of Massachusetts Medical School (UMMS), which received a contract from TMC to perform this work.

## Authors' contributions

The Euro-STAT Steering Committee was responsible for the design and scientific and ethical conduct of the study, including assurance that appropriate measures for the protection of the participants, including hospitals, physicians and patients, were instituted and followed consistently. AV, JLV, DPG, FAA and JMG were fully involved in the design and conduct of the study, had access to all study analyses, drafted and edited the paper and agreed upon its final contents.

## Euro-STAT Steering Committee members

Co-Chairs: Didier Payen de la Garanderie, Paris, France; Alain Vuylsteke, Cambridge, UK. Members: Frederick A Anderson, Worcester, MA, USA; Antonio Artigas, Sabadell, Spain; Jean-Daniel Chiche, Paris, France; A Raffaele De Gaudio, Florence, Italy; Salvatore Di Somma, Rome, Italy; Joel M Gore, Worcester, MA, USA; Christopher B Granger, Durham, NC, USA; Michael J Hiesmayr, Vienna, Austria; Andreas Hoeft, Bonn, Germany; Bengt Jönsson, Stockholm, Sweden; Andreas Koster, Berlin, Germany; Thomas Lüscher, Zurich, Switzerland; Jan Ostergren, Stockholm, Sweden; W Frank Peacock, Cleveland, OH, USA; Kees Polderman, Utrecht, The Netherlands; David Smith, Southampton, UK; Jean-Louis Vincent, Brussels, Belgium.
